# Should Topical Ice Slush Be Used Routinely in Cardiac Surgery? Topical Ice Slush in Cardiac Surgery

**DOI:** 10.3390/jcm14196980

**Published:** 2025-10-02

**Authors:** Osman Fehmi Beyazal, Suleyman Yazici, Zeki Temizturk, Cemalettin Aydin, Hasan Tezcan, Selman Sadi Citak, Nihan Kayalar, Mehmed Yanartas

**Affiliations:** Department of Cardiovascular Surgery, Basaksehir Cam and Sakura City Hospital, Basaksehir G-434 Street No: 2L, Istanbul 34480, Turkey; drsuleymanyazici@gmail.com (S.Y.); zekitmztrk5806@gmail.com (Z.T.); cemalettinaydin1@hotmail.com (C.A.); hasan.tezcan44@hotmail.com (H.T.); dr.selmansadi@gmail.com (S.S.C.); nkayalar@hotmail.com (N.K.); myanartas@yahoo.com (M.Y.)

**Keywords:** ice slush, hypothermia, diaphragm elevation, cardiac surgery, phrenic nerve

## Abstract

**Background:** The aim of this study is to investigate the effect of topical ice slush on cardiac protection in patients undergoing cardiac surgery and to analyze its potential side effects. **Methods:** Between 2023 and 2024, 890 patients who underwent cardiac surgery were evaluated. The patients were divided into two groups: Group A (*n* = 549), assigned ice slush(+), and Group B (*n* = 341), assigned ice slush(−). Echocardiographic findings, laboratory parameters, arterial blood gas findings, inotrope requirements, and postoperative outcomes were compared. Patients with a left internal thoracic artery were excluded from this study, and new subgroups were created as follows: Group C (*n* = 235), assigned ice slush(+), and Group D (*n* = 111), assigned ice slush(−). Chest radiography and diaphragm elevations (DEs) were compared at the 12-month follow-up. **Results:** No significant differences were found between the groups in terms of demographic characteristics, comorbidities, and operative data. The postoperative echocardiographic findings and ABG findings were similar. The inotrope requirement was higher in Group A. Postoperative day 1 Troponin T was higher in Group A than in Group B (median: 561–473 ng/mL, *p* = 0.01). The postoperative outcomes were similar between the groups, except that the intubation duration was longer in Group A. In the 1st postoperative week, 199 (36.2%) patients in Group A and 127 (37.2%) patients in Group B had DE. In the 12th month postoperation, 20 (3.6%) patients in Group A and 12 (3.5) patients in Group B had DE. Although not statistically significant, the incidence of DE was higher in Group C than in Group D in the early postoperative period only. **Conclusions:** We found no additional cardioprotective benefit from the use of topical ice slush in cardiac surgery. The intubation time was longer in patients with topical ice slush than in patients without it. Our results suggest that the routine use of topical ice slush in cardiac surgery is not necessary and that it has potential adverse effects.

## 1. Introduction

Since the advent of cardiac surgery, surgeons have known that cooling the myocardium protects against cellular damage [1]. Cold cardioplegia solutions can be used for this purpose, and topical ice slush is also used around the heart and in the pericardial space during cardioplegic arrest. Topical ice slush cools the heart in addition to cold cardioplegia during the time between intermittent cardioplegias. However, with the development of technology and perfusion techniques, the necessity of ice slush has become a matter of debate. Alassar et al. reported no evidence of any additional cardioprotective benefit from topical cooling [2]. Apart from the debate regarding the benefits of topical ice slush, its potential harms have also been investigated. Aguirre et al. reported that phrenic nerve injury is common in cardiac surgery involving slush [3]. Kadan et al. also reported that topical hypothermia had adverse effects on inflammatory markers and postoperative morbidities [4].

Although not used in all centers, ice slush is still routinely used in many centers and by many surgeons. Civelek et al. investigated diaphragm elevations in 888 patients and reported that topical ice slush was routinely used during surgical procedures [5]. In our clinic, topical ice slush is used according to the surgeon’s preference. Although studies have investigated this subject, the current guidelines do not provide definitive information on whether topical ice slush should be used routinely. In addition, studies on the protective and adverse effects of topical ice slush have included relatively few patients and parameters. Therefore, we designed this study to investigate the effect of cooling the heart with topical ice slush on cardiac protection in patients undergoing cardiac surgery, to analyze its potential side effects, and to confirm the necessity of its routine use in cardiac surgery.

## 2. Methods

This study was a retrospective single-center observational study with a total of 890 patients. All patients over the age of 18 who underwent cardiac surgery at the Cardiovascular Surgery Clinic of Istanbul Basaksehir Cam and Sakura City Hospital between January 2023 and May 2024 were included. The following patients who underwent beating heart surgery were excluded from this study: patients who did not receive hypothermia, aortic dissections, total arch surgery, or reoperations, and patients who received extracorporeal membrane oxygenation in the perioperative period. The patients who met the inclusion criteria were first divided into two groups: Group A (*n* = 549) included those who received topical ice slush, and Group B (*n* = 341) included those who did not receive topical ice slush. Then, the patients who used the left internal thoracic artery (LITA) were excluded from this study, and new subgroups were created as follows: Group C (*n* = 235) (patients without LITA, received topical ice slush) and Group D (*n* = 111) (patients without LITA, did not receive topical ice slush). In our clinic, surgeons prefer the use of topical ice. Some surgeons use it routinely, whereas some do not, and some use it for patients other than those who did not receive LITA. In Group A patients, topical ice slush was used in the pericardial space once, after the cross-clamp was placed following hypothermia. It was removed by aspiration after the end of cardioplegia.

All the patients’ medical records were reviewed to obtain basic demographic characteristics; medical histories; European System for Cardiac Operative Risk Evaluation (EuroSCORE II); New York Heart Association (NYHA) stages; preoperative and postoperative first week transthoracic echocardiographic (TTE) findings; pulmonary function tests’ preoperative, 1st postoperative day, and 1st postoperative month laboratory parameters; arterial blood gas (ABG) findings on the morning of the operation; cross clamp (XCL) 30 min after the operation and on the 1st postoperative day; preoperative and postoperative 1st week, 1st month, 3rd month, 6th month, and 12th month chest X-ray findings; surgical procedure details; XCL times; cardiopulmonary bypass (CPB) times; near-infrared spectroscopy (NIRS) findings; inotrope doses; amount of bleeding; blood product use; and postoperative complications (diaphragm elevation (DE), thoracentesis need, postoperative exploration, postoperative atrial fibrillation (POAF), cerebrovascular accident (CVA), continuous renal replacement therapy (CRRT) need, deep sternal wound infection (DSWI), percutaneous coronary intervention (PCI) need, tracheostomy, total mortality, early mortality (<30 days), late mortality (≥30 days), intubation time, intensive care unit (ICU) stay, hospital stay). First, these parameters were compared between Groups A and B. Then, to prevent the possible negative effect of LITA harvest on DE, the newly created subgroups were compared in terms of DE.

Diagnostic methods such as ultrasonography and fluoroscopy are used to define DE; however, because this study was retrospective, DE was determined using chest X-ray images. Pathologies that could mimic DE, such as pleural effusion, atelectasis, and hematoma, were carefully examined and distinguished. In suspicious cases, a differential diagnosis was made with thorax computed tomography images. The literature contains different definitions of DE for chest X-rays. In our study, the definition of Draeger et al. was used [6], where right DE was considered as a difference of >40 mm between the right and left diaphragm heights, and left DE was considered as the left diaphragm being at the same height or higher than the right. However, since this definition may be insufficient in the case of DE on both sides, the elevation of one costal space on the left side and two costal spaces on the right side was also considered when compared with the preoperative chest X-ray used by Nazer et al. [7].

This study was approved by the Istanbul Basaksehir Cam and Sakura City Hospital Ethics Committee (decision no.: 2025-191, 18 June 2025) and was conducted in accordance with the Declaration of Helsinki. Artificial intelligence technologies were not used in the production of the submitted work.

## 3. Statistics

The data were analyzed with SPSS software version 20.0 (IBM, Armonk, NY, USA). Continuous variables are presented as the minimum, maximum, median, and interquartile range. Categorical variables are expressed as numbers and percentages. The normality of data distributions was assessed using the Kolmogorov–Smirnov test. For numerical variables, differences between the patients and controls were tested using the *t*-test for parametric data or the Mann–Whitney U test for nonparametric data. Categorical variables were analyzed using the Pearson χ^2^ test and Fisher’s exact test for parametric and nonparametric data, respectively. Multivariate logistic regression analysis was performed to determine the factors affecting DE. The level of statistical significance was set at *p* < 0.05.

## 4. Results

The patients’ demographics, comorbid diseases, medications, and TTE findings are shown in Table 1. The mean age was 59.2 ± 10.9 years, and 644 (72.4%) of the patients were male. The mean follow-up period was 589.6 ± 196.9 days (median: 614, 1–861 days). No difference was found between the groups in terms of basic demographic characteristics and comorbid diseases. Preoperative pulmonary function tests were similar between the groups. No difference was found between the groups in terms of the EuroSCORE II and NYHA stages. There was no difference in the preoperative TTE findings in terms of ejection fraction (EF) or tricuspid annular plane systolic excursion (TAPSE). No difference was found between Group A and Group B in terms of postoperative EF and TAPSE values.

A comparison of the operation data and ABG findings is shown in Table 2. No difference was found between the groups in terms of emergency surgery rates. All cardiac operation types, except those meeting the exclusion criteria, were included in this study, and the patients were classified and compared as isolated coronary artery bypass grafting (CABG), valvular surgery, and aortic surgery. Accordingly, no difference was found between the groups in terms of isolated CABG, valvular surgery, aortic surgery, and concomitant procedures. However, LITA use was significantly lower in the patient group that received topical ice slush compared to the group that did not (314 (57.2%) and 230 (67.4), respectively, *p* = 0.002). The number of bypassed grafts was similar between the groups. CPB times were higher in Group A than in Group B, but there was no statistical difference (median 140 min and 132 min, respectively, *p* = 0.27). However, XCL times were significantly higher in Group A than in Group B (median 94 min and 83 min, respectively, *p* = 0.002). No difference was found between the groups for both sides in terms of the NIRS findings before XCL and 30 min after XCL. No difference was found between the groups in terms of the ABG findings (pH, PaCO_2_, and lactate) on the morning of the operation. No difference was found between the groups in terms of the ABG findings (pH, PaCO_2_, hematocrit, and lactate) 30 min after XCL. No difference was found between the groups in terms of the ABG findings (pH, PaCO_2_, and lactate) on postoperative day 1.

A comparison of inotrope doses, bleeding amount, and blood product usage is shown in Table 3. There was no difference between the groups in terms of epinephrine and norepinephrine requirements: <0.1 mcg/kg/min and ≥0.1 mcg/kg/min. There was no difference between the groups in terms of dopamine ≥ 10 mcg/kg/min and dobutamine < 10 mcg/kg/min requirements, but dopamine < 10 mcg/kg/min and dobutamine ≥ 10 mcg/kg/min requirements were higher in Group A than in Group B (32 (5.8%) vs. 17 (5%), *p* = 0.007, 97 (17.7%) vs. 41 (12%), *p* = 0.02, respectively). There was no difference between the groups in terms of total bleeding amount. There was no difference between the groups in terms of intraoperative red blood cells (RBCs), fresh frozen plasma (FFP), and platelet suspension use. However, postoperative RBC and FFP use were higher in Group A than in Group B.

A comparison of laboratory parameters between groups is shown in Table 4. No difference was found between Group A and Group B in all preoperative parameters. While the 1st postoperative day white blood cells (WBCs), sodium, and aspartate aminotransferase values were higher in Group A than in Group B, the platelet values were lower. The postoperative day 1 Troponin T value was higher in Group A than in Group B (median: 561 ng/mL and 473 ng/mL, respectively, *p* = 0.01). No difference was found between the groups in terms of other 1st postoperative day values (hematocrit, urea, creatinine, alanine aminotransferase, potassium, CRP values). The CK-MB values were also higher in Group A than in Group B, but no statistical difference was found. No difference was found between the groups in terms of all these parameters in the 1st postoperative month.

A comparison of the postoperative outcomes is shown in Table 5 and Table 6. Preoperative DE was detected in 14 (2.6%) patients in Group A and 11 (3.2%) patients in Group B. No difference was found between the groups in terms of preoperative, 1st postoperative week, 1st postoperative month, 3rd postoperative month, 6th postoperative month, and 12th postoperative month DE. In the 1st postoperative week, 199 (36.2%) patients in Group A and 127 (37.2%) patients in Group B had DE. In the following months, these DE rates continued to decrease gradually. In the 12th month postoperation, 20 (3.6%) patients in Group A and 12 (3.5) patients in Group B had DE. Additionally, there was no difference between the groups in terms of postoperative exploration, CVA, CRRT need, POAF, DSWI, gastrointestinal bleeding, PCI need, pleural effusion drainage requirement, tracheostomy need, and mortality rates. Intubation time was significantly longer in Group A than in Group B (mean 26.3 h–20.9 h, respectively, *p* = 0.04). However, there was no difference between the groups in terms of ICU stay and hospital stay.

Multivariate regression analysis was performed to determine the effect of LITA harvest on DE, and the results are shown in Table 7. No significant association was found between gender, age, diabetes mellitus (DM), chronic obstructive pulmonary disease (COPD), and topical ice slush. However, a significant association was found between LITA harvest and DE (*p* = 0.01, OR = 1.468 [95% CI, 1.081–1.995]).

**Figure 1:**
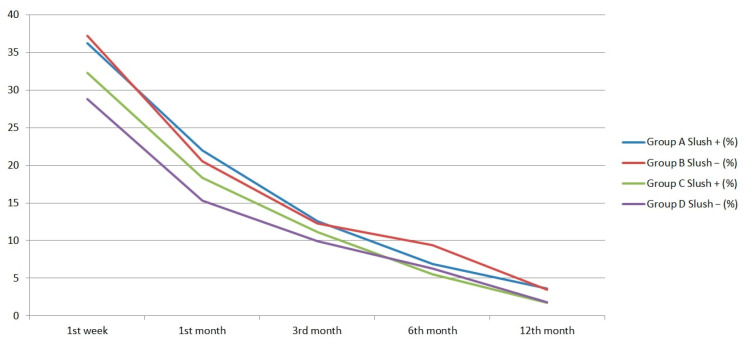
Diaphragm elevation rates in all groups over 12 months.

A comparison of DE and the subgroups after patients with LITA were excluded is shown in Table 8. No statistically significant difference was found between the groups during the entire follow-up period in terms of DE. A graphical display of DE rates in all groups according to time is shown in Figure 1. Although not statistically significant, the incidence of DE was higher in Group C than in Group D (Figure 1) in the early postoperative period only (1st week, 1st month, and 3rd month). During the 1st postoperative week, DE was detected in 76 (32.3%) patients in Group C and in 32 (28.8%) patients in Group D. During the 12th postoperative month, DE was detected in four (1.7%) patients in Group C and two (1.8%) patients in Group D.

## 5. Discussion

Compared to the early years of cardiac surgery, the more effective cardioplegic solutions of today have resulted in better myocardial protection. However, in addition to lowering body temperature, many surgeons also apply a topical hypothermia method, believing that it is beneficial for myocardial protection. Today, many surgeons still use this method to achieve a cardioprotective effect. However, studies have shown that this cardioprotective effect is not significant [1,2,8]. In addition, potential adverse effects on the diaphragm have been reported [3,5,7,9,10,11]. However, the current guidelines do not contain definitive information about its routine use. Most previous studies included a small number of patients. Braathen et al. reported no additional cardioprotective effect of ice slush in a study including 60 patients [1]. Kadan et al. studied 50 patients and reported a negative effect of topical hypothermia on inflammatory markers [4]. Canbaz et al. studied 78 patients and reported that ice slush played a role in phrenic nerve damage [9]. In the study by Civelek et al., 74 patients with and without ice slush were compared, and DE was found to be higher in those with ice slush [5]. Nazer et al. studied 20 patients and reported that topical ice slush was associated with phrenic nerve damage [7]. In our study, we conducted a detailed analysis of 890 patients and several parameters to investigate the cardioprotective effect of topical ice slush and to evaluate its effects on the respiratory system. We found that topical ice slush did not provide an additional cardioprotective effect in cardiac surgery, and the incidence of DE was higher in the early postoperative period, although it was not statistically significant.

In this study, the basic demographic characteristics, comorbidities, echocardiographic findings, and preoperative laboratory parameters of the patient groups were similar. There was no difference in EuroSCORE II, NYHA stages, and pulmonary function tests. In addition, the types of operations were similar, as listed in Table 2. Based on these results, we can say that a similar patient group was compared. The main difference between the groups was the use of topical ice slush according to the surgeon’s preference. When the effects on myocardial protection were examined, the preoperative EF and TAPSE values were similar in both groups. No significant difference was found in the TTE findings (EF and TAPSE) in the first postoperative week. In addition, no difference was found between the groups in the ABG findings (pH, PaCO_2_, lactate) measured 30 min after topical ice slush administration and on the first postoperative day. In addition, inotrope requirements were compared with separate doses for each inotrope type, and the patients who did not use topical ice slush had similar or less inotrope requirements. Finally, the laboratory parameters on the first postoperative day also included similar or better results in terms of all parameters in the group without ice slush. No difference was found between the groups in terms of all parameters in the first postoperative month. No difference was found in terms of postoperative outcomes and mortality rates. Based on these results, the use of topical ice slush does not provide an additional benefit in myocardial protection. Apart from this, arrhythmias may increase with hypothermic myocyte damage [4]. However, in our study, no difference was found between the groups in terms of new-onset POAF. Unlike other studies on myocardial protection, in this study, we performed a detailed comparison to evaluate myocardial functions. Furthermore, not only Troponin T and CK-MB results, but also postoperative EF and TAPSE, were used to evaluate right and left ventricular functions, and intraoperative and postoperative ABG findings, inotrope doses and types, and postoperative outcomes were compared in detail. Therefore, this study presents more detailed results than other studies by showing that ice slush does not provide a significant cardioprotective effect.

A noteworthy result of this study was the statistically significant increase in Troponin T values and statistically insignificant increase in CK-MB values in patients treated with topical ice slush. In our study group, XCL times were higher in Group A than in Group B. This early elevation in Troponin T may be related to the high XCL times, as well as myocardial and epicardial tissue damage caused by ice slush. In fact, Nikas et al. reported that myocardial and epicardial injury may occur with ice slush [8]. However, it is not possible to make a clear distinction based on these findings. We also found that this difference disappeared in the first month postoperatively, and similar results were obtained. Similarly, in our detailed inotrope comparison, inotropes were similar or lower in the group that did not receive ice slush. This may also be related to the higher XCL times or may be an indicator of myocardial damage in the ice slush group, but this distinction cannot be established based on these results. Troponin T values were similar at 1 month postoperatively, and we found no difference in TTE findings at 1 week postoperatively. However, it would be beneficial to investigate the potential long-term adverse effects of ice slush on the myocardium using randomized controlled trials. However, although the myocardial damage caused by ice slush is not definite, we suspect that it does not have a significant cardioprotective effect.

Another important point about ice slush is its potential disadvantages. Apart from the myocardium, it can damage the phrenic nerve and cause spasms in the LITA. In fact, for this reason, some surgeons do not prefer ice slush in patients with LITA. In our study, although the operation types were similar, there was a significant difference in terms of LITA usage rates. The main reason for this is that some surgeons do not prefer slush in cases where LITA is used. The relationship between LITA use and phrenic nerve damage and DE is well known [5]. The phrenic nerve has a close anatomical relationship with the LITA. The phrenic nerve originates from the fourth cervical nerve root, receives contributions from the third and fifth cervical nerve roots, passes from the lateral to the medial aspect of the scalenus anterior muscle, and proceeds downward. The right phrenic nerve crosses the right internal thoracic artery (RITA) from lateral to medial, proceeds from the right side of the innominate vein and superior vena cava, passes over the right atrium and inferior vena cava, and reaches the diaphragm. On the left side, it passes through the scalenus anterior muscle and the LITA; then, it passes lateral to the aortic arch and proceeds along the left lateral aspect of the pericardium and reaches the diaphragm [3]. The phrenic nerve can be injured during LITA harvest due to its close anatomical relationship with the LITA; cautery damage can occur, and the phrenic nerve can be injured during pericardiectomy to tunnel the LITA pedicle. Phrenic nerve dysfunction can also be seen by ligating arteries that provide blood flow to the phrenic nerve originating from the LITA or the subclavian artery. Phrenic nerve damage can be due to demyelination and direct contact in patients treated with ice slush. In contrast, Canbaz et al. conducted a study on 78 patients and detected no phrenic nerve damage in patients who underwent beating surgery using LITA [9]. We also performed multivariate regression analysis in our study and found that LITA harvest alone was associated with DE. However, we did not find a significant association with age, gender, chronic obstructive pulmonary disease, or ice slush use. Similarly, according to the logistic regression analysis in the study by Merino-Ramirez et al., age, gender, and DM were not risk factors for phrenic nerve damage [12]. However, to better evaluate the effect of ice slush on DE and to prevent the effect of LITA use on respiratory complications, we excluded all patients in whom LITA was used and re-compared them with new subgroups in terms of DE.

In the first comparison, the frequency of DE in Group A in the first postoperative week was 36.2%, while it was 37.2% in Group B. In the subgroups, the frequency of DE in Group C was 32.3% and 28.8% in the first postoperative week. Although LITA harvest is an important risk factor for DE, we found a considerable amount of DE in patients without LITA use. Although there was no statistically significant difference, we observed that the frequency of DE in Group C was higher than in Group D in the early postoperative period (up to the first 3 months) (Figure 1). The incidence of DE after cardiac surgery is between 1.2% and 60% in the literature [3]. While the most important cause of DE before cardiac surgery was congenital, acquired DE plays an important role after cardiac surgery. One reason for this wide range is that the definition of DE differs across studies [4,5,6,7,8]. The incidence in our study is also consistent with these rates; however, the vast majority of these DEs recover over time. In our study, at 12 months, we observed 3.6% DE in Group A, 3.5% in Group B, 1.7% in Group C, and 1.8% in Group D. In the study by Civelek et al., 85% recovery was reported at 1 year [5]. In our study, 96.4% recovery was observed. However, we found that the incidence of DE was low in patients without LITA and that recovery rates were better after 12 months. We observed that 98.3% of the patients without LITA recovered. Axons generally regenerate at a constant rate of approximately 1 mm per day [13], and the phrenic nerve’s length of 24.6 ± 1.7 cm on the right and 30.6 ± 1.8 cm on the left is consistent with this healing process [14]. Therefore, this recovery period is to be expected.

Another important point for DE is the side on which it is located. In the entire patient group, 277 (31.1%) patients had left DE in the first postoperative week, while 49 (5.5%) patients had right DE. In the subgroup, 81 (23.4%) patients had left DE in the first postoperative week, while 27 (7.8%) patients had right DE. The clinical course is more severe with bilateral DE, but bilateral DE was not detected in any patient in our study group. The reason for the more frequent damage to the left phrenic nerve may be associated with LITA harvest, but the frequency of DE on the left side is also significantly higher in patients without LITA. The main reason for this may be that the left ventricle is the main target of myocardial protection, and ice slush is often applied around the left ventricle on the left side of the pericardium. Therefore, the right phrenic may be protected more frequently. In addition, factors such as the duration that the ice slush remains in the pericardial space and whether it is given again during each cardioplegia are also important at this point.

Finally, the presence of DE and the increase in pulmonary complications associated with it increase the length of hospital stay and cost [8]. Additionally, diaphragm dysfunction may delay successful extubation [15]. No difference was found between the groups in terms of pleural effusion drainage and tracheostomy requirements. No difference was found between the groups in terms of ICU stay and hospital stay. However, intubation time was found to be significantly higher in Group A than in Group B. Ice slush may not be the only reason for this difference. However, although not statistically significant, higher DE rates in the early postoperative period may have had an effect. However, comparing this with patients with and without DE in randomized studies will yield more accurate results.

## 6. Limitations

The first limitation of this study is that it is retrospective and single-centered. Although the groups had similar baseline characteristics, the results may have been influenced by unmeasured confounding variables. Second, chest X-ray images were examined for the diagnosis of DE. Ultrasonography, fluoroscopy, or electrophysiological studies could not be performed. Third, the long-term results of some factors, such as the TTE findings, could not be compared. No difference was observed in myocardial protection during the early period, but it would not be correct to generalize this to the mid-term results. Topical ice slush use may differ between centers or surgeons. In some centers, ice slush is given during every cardioplegia, while in others, it is applied only during the first cardioplegia. In addition, the type of cardioplegia is important. While it is given at every dose in blood cardioplegia, a single dose application is often sufficient in del Nido cardioplegia. Repeated application of ice slush may change the risk of DE. However, it may be useful to investigate this in future studies.

## 7. Conclusions

In this study, we found that the use of topical ice slush provided no additional cardioprotective benefit in cardiac surgery. We found that the intubation time was longer in patients who received topical ice slush than in patients who did not. We also found that the incidence of diaphragm elevation in the early postoperative period was higher in patients who received topical ice slush than in patients who did not, although there was no statistically significant difference. Based on these results, we suspect that the routine use of topical ice slush in cardiac surgery is not necessary and that it has potential adverse effects. However, future large-scale randomized controlled trials are needed to investigate this topic further.

## Figures and Tables

**Table 1:** Comparison of patient demographics, comorbidities, medications, and echocardiographic findings between Group A and Group B.

	Group A (Slush +) (*n* = 549)			Group B (Slush −) (*n* = 341)			
	Min–Max or *n* (%)	Median (Mean)	IQR	Min–Max or *n* (%)	Median (Mean)	IQR	*p*
**Demographic data**							
Gender male	392 (71.4)			252 (73.9)			0.41
Age (years)	20–90	61	14	19–86	59	16	0.18
Height (cm)	140–195	168	12	140–196	169	13	0.56
Weight (kg)	42–127	78	18	38–130	80	20	0.22
Body surface area (kg/m^2^)	1.33–2.42	1.88	0.22	1.22–2.38	1.89	0.25	0.24
Body mass index (m^2^)	17.1–48.8	27.7	6.1	15.6–55.6	28	6.5	0.55
**Comorbid diseases**							
Diabetes Mellitus	230 (41.9)			152 (44.6)			0.43
Hypertension	298 (54.3)			190 (55.7)			0.67
Chronic obstructive pulmonary disease	46 (8.4)			33 (9.7)			0.50
Cerebrovascular accident	44 (8)			23 (6.7)			0.48
Chronic renal failure	25 (4.6)			19 (5.6)			0.49
Peripheral artery disease	30 (5.5)			15 (4.4)			0.48
Malignancy history	26 (4.7)			17 (5)			0.86
Thyroid disorder	50 (9.1)			32 (9.4)			0.89
Rheumatic disease	9 (1.6)			10 (2.9)			0.19
Operation with dual antiplatelet	11 (2)			8 (2.3)			0.73
Immunosuppressive drug	5 (0.9)			6 (1.8)			0.20
FEV1/FVC (%)	35.4–99.3	83.1	10.9	40.2–99.4	82.2	10.3	0.96
EuroSCORE II	0.5–45	2 (3)	2	0.6–28	2 (2.6)	2	0.65
Preop NYHA stage	1–4	2 (2.07)	0	1–4	2 (2.06)	0	0.77
**Echocardiographic findings**							
Preop ejection fraction (%)	20–65	60	10	20–65	60	10	0.22
Preop TAPSE (mm)	14–41	23	5	13–36	23	5	0.17
Postop ejection fraction (%)	20–65	55	10	25–65	55	10	0.51
Postop TAPSE (mm)	5–31	16	5	6–27	16	6	0.57

IQR: interquartile range, FEV1: Forced expiratory volume in one second, FVC: Forced vital capacity, EuroSCORE II: The European System for Cardiac Operative Risk Evaluation, NYHA: The New York Heart Association, TAPSE: tricuspid annular plane systolic excursion.

**Table 2:** Comparison of operative data and arterial blood gas between Group A and Group B.

	Group A (Slush +) (*n* = 549)			Group B (Slush −) (*n* = 341)			
	Min–Max or *n* (%)	Median (Mean)	IQR	Min–Max or *n* (%)	Median (Mean)	IQR	*p*
**Operative data**							
Emergency surgery	26 (4.7)			12 (3.5)			0.38
Isolated CABG	352 (64.1)			208 (61)			0.34
Isolated valvular surgery	116 (21.1)			67 (19.6)			0.59
Aortic surgery	40 (7.3)			19 (5.6)			0.31
Concomitant procedures	86 (15.7)			63 (18.5)			0.27
LITA usage	314 (57.2)			230 (67.4)			**0.002**
The number of grafts	0–7	2 (2.1)	3	0–6	3 (2.2)	4	0.08
Cross-clamp time (min)	15–323	94	61	3–288	83	58	**0.002**
Cardiopulmonary bypass time (min)	36–418	140	65	46–391	132	67	0.27
Before XCL NIRS right (%)	33–99	62	12	32–85	62	12	0.52
Before XCL NIRS left (%)	30–93	62	12	31–83	62	11	0.59
After XCL NIRS right (%)	32–87	59	10	29–77	59	9	0.46
After XCL NIRS left (%)	23–95	59	9	31–83	58.5	10	0.98
**Arterial blood gas**							
Preop pH	7.05–7.59	7.41	0.05	7.28–7.54	7.42	0.05	0.67
Preop PaCO_2_ (mmHg)	22–78	38	6	23–90	38	6	0.42
Preop lactate (mmol/L)	0.3–15	1 (1.1)	0.6	0.4–4.9	1 (1.1)	0.6	0.73
Cross clamp 30th minute pH	7.21–7.61	7.41	0.06	7.30–7.58	7.40	0.07	0.14
Cross clamp 30th minute hematocrit (%)	16–42	26.7	6	16–43	26.2	6	0.94
Cross clamp 30th minute PaCO_2_ (mmHg)	20–58	38.2	5	25–49	38.2	6	0.50
Cross clamp 30th minute lactate (mmol/L)	0.3–15	1.8 (2.05)	1.1	0.1–7.4	1.8 (2.03)	1.5	0.87
Postop 1st day pH	7.12–7.60	7.43	0.06	7.29–7.58	7.43	0.06	0.66
Postop 1st day PaCO_2_ (mmHg)	20–99	37.9	6	22–49	37.3	5	0.10
Postop 1st day lactate (mmol/L)	0.6–28	2.1 (2.7)	1.8	0.6–18	2 (2.4)	1.6	0.11

IQR: interquartile range, CABG: coronary artery bypass grafting, LITA: left internal thoracic artery, XCL: cross-clamp, NIRS: near-infrared spectroscopy.

**Table 3:** Comparison of inotrope doses, bleeding, and blood products between Group A and Group B.

	Group A (Slush +) (*n* = 549)			Group B (Slush −) (*n* = 341)			
	Min–Max or *n* (%)	Median (Mean)	IQR	Min–Max or *n* (%)	Median (Mean)	IQR	*p*
**Inotrope doses**							
Postop epinephrine < 0.1 mcg/kg/min	2 (0.4)			1 (0.3)			0.67
Postop epinephrine ≥ 0.1 mcg/kg/min	26 (4.7)			11 (3.2)			0.27
Postop norepinephrine < 0.1 mcg/kg/min	23 (4.2)			11 (3.2)			0.46
Postop norepinephrine ≥ 0.1 mcg/kg/min	65 (11.8)			28 (8.2)			0.08
Postop dopamine < 10 mcg/kg/min	32 (5.8)			7 (2.1)			**0.007**
Postop dopamine ≥ 10 mcg/kg/min	46 (8.4)			17 (5)			0.055
Postop dobutamine < 10 mcg/kg/min	171 (31.1)			98 (28.7)			0.44
Postop dobutamine ≥ 10 mcg/kg/min	97 (17.7)			41 (12)			**0.02**
**Bleeding and blood products**							
Intraop red blood cells using	0–13	0 (1.03)	2	0–14	0 (1.05)	2	0.97
Intraop fresh frozen plasma using	0–4	0 (0.4)	0	0–4	0 (0.3)	0	0.17
Intraop platelet suspensions	0–6	0 (0.2)	0	0–4	0 (0.1)	0	0.13
Postop red blood cells using	0–16	0 (1.1)	2	0–9	0 (0.88)	1	**0.01**
Postop fresh frozen plasma using	0–9	1 (0.99)	2	0–6	0 (0.59)	1	**<0.001**
Postop platelet suspensions	0–6	0 (0.1)	0	0–9	0 (0.14)	0	0.14
Amount of bleeding (mL)	100–4060	700	500	100–4050	700	425	0.39

**Table 4:** Comparison of laboratory parameters between Group A and Group B.

	Group A (Slush +) (*n* = 549)		Group B (Slush −) (*n* = 341)		
	Min–Max or *n* (%)	Median (IQR)	Min–Max or *n* (%)	Median (IQR)	*p*
**Preop laboratory parameters**					
White blood cells (10^9^/L)	2.6–29.1	8.1 (3.2)	2–21.1	8.5 (3)	0.92
Hematocrit (%)	22–53	41 (7)	21–57	41 (7)	0.98
Platelets (10^9^/L)	88–772	243 (96)	46–670	253 (94)	0.34
Urea (mg/dL)	8.2–272	34.8 (15.7)	9.3–122.4	34 (16.8)	0.57
Creatinine (mg/dL)	0.4–9.02	0.91 (0.3)	0.27–10.9	0.92 (0.3)	0.95
Sodium (mEq/L)	122–149	139 (4)	120–149	139 (4)	0.69
Potassium (mEq/L)	2.2–5.9	4.4 (0.6)	2.9–6.0	4.34 (0.6)	0.10
Alanine aminotransferase (IU/L)	1–338	18 (12)	3–172	17 (13)	0.90
Aspartate aminotransferase (IU/L)	4–340	20 (10)	5–431	20 (11)	0.43
C-reactive protein (mg/dL)	0.2–195	3.6 (8)	0.1–270	3.8 (8.5)	0.71
HbA1c (mmol/mol)	4.4–16.2	6 (1.4)	4.2–14.3	6 (1.5)	0.76
Troponin T (ng/mL)	1–1994	15 (23)	1–1860	15 (24)	0.53
CK-MB (IU/L)	0.1–353	1.5 (1.2)	0.1–37.5	1.5 (1.3)	0.67
**Postop 1st day laboratory parameters**					
White blood cells (10^9^/L)	5.5–59.8	16.8 (9)	4.4–44.3	15.7 (8.6)	**0.02**
Hematocrit (%)	19–60	28 (5)	13–44	29 (6)	0.06
Platelets (10^9^/L)	33–550	170 (80)	39–496	182 (82)	**0.01**
Urea (mg/dL)	5.3–200	41 (18)	16–170.7	41.3 (18.9)	0.57
Creatinine (mg/dL)	0.04–5.7	1.27 (0.47)	0.4–7.82	1.22 (0.47)	0.87
Sodium (mEq/L)	126–163	143 (4)	133–158	142 (4)	**0.001**
Potassium (mEq/L)	2.7–8.9	4.26 (0.73)	3.18–5.76	4.3 (0.66)	0.75
Alanine aminotransferase (IU/L)	1–1221	24 (18)	2–704	22 (15)	0.07
Aspartate aminotransferase (IU/L)	11–3962	62 (43)	8–7031	57.5 (38)	**0.006**
C-reactive protein (mg/dL)	6.2–304	36.6 (25.3)	4.4–324	37.8 (24.5)	0.66
Troponin T (ng/mL)	11–11,306	561 (701)	24 (2301)	473 (423)	**0.01**
CK-MB (IU/L)	0.1–1373	10.6 (24.2)	0.1–300	9 (22.4)	0.70
**Postop 1st month laboratory parameters**					
White blood cells (10^9^/L)	1.4–111	8.2 (3.3)	2.6–21.3	8.1 (3.5)	0.48
Hematocrit (%)	23–61	35 (6)	20–45	34 (6)	0.53
Platelets (10^9^/L)	27–922	343 (167)	9–819	355 (161)	0.52
Urea (mg/dL)	10.4–177.8	33.4 (18.5)	10–134	33.1 (16.4)	0.93
Creatinine (mg/dL)	0.38–7.48	0.93 (0.31)	0.39–11.2	0.96 (0.35)	0.29
Sodium (mEq/L)	120–150	138 (4)	126–147	138 (4)	0.21
Potassium (mEq/L)	3.05–6	4.52 (0.67)	3.15–5.75	4.57 (0.61)	0.52
Alanine aminotransferase (IU/L)	3–5703	16 (12)	4–248	17 (14)	0.11
Aspartate aminotransferase (IU/L)	4–7000	16 (9)	7–128	16 (9)	0.89
C-reactive protein (mg/dL)	0.3–195.3	19.4 (38.7)	2–418	18.6 (33.4)	0.60
Troponin T (ng/mL)	10–263	33.5 (49)	9–1387	29.5 (44)	0.57
CK-MB (IU/L)	0.1–43.1	1.4 (1)	0.1–8.5	1.4 (1)	0.37

**Table 5:** Comparison of postoperative outcomes between Group A and Group B.

	Group A (Slush +) (*n* = 549) *n* (%)	Group B (Slush −) (*n* = 341) *n* (%)	
Preop diaphragmatic elevations (DE)	14 (2.6)	11 (3.2)	0.55
Postop 1st week DE	199 (36.2)	127 (37.2)	0.76
Postop 1st month DE	121 (22)	70 (20.5)	0.59
Postop 3rd month DE	69 (12.6)	42 (12.3)	0.91
Postop 6th month DE	38 (6.9)	32 (9.4)	0.11
Postop 12th month DE	20 (3.6)	12 (3.5)	0.54
Postoperative exploration	52 (9.5)	25 (7.3)	0.26
Cerebrovascular accident	16 (2.9)	5 (1.5)	0.16
Continuous renal replacement therapy	26 (4.7)	14 (4.1)	0.65
Postop atrial fibrillation	104 (18.9)	65 (19.1)	0.96
Deep sternal wound infection	21 (3.8)	11 (3.2)	0.64
Gastrointestinal bleeding	2 (0.4)	3 (0.9)	0.28
Percutaneous coronary intervention	3 (0.5)	1 (0.3)	0.50
Pleural effusion drainage	28 (5.1)	24 (7)	0.23
Tracheostomy	9 (1.6)	6 (1.8)	0.89
Total mortality	32 (5.8)	19 (5.6)	0.87
Early mortality (<30 days)	24 (4.4)	15 (4.4)	0.98
Late mortality (≥30 days)	8 (1.5)	4 (1.2)	0.48

**Table 6:** Comparison of intubation time and hospital stay between Group A and Group B.

	Group A (Slush +) (*n* = 549)			Group B (Slush −) (*n* = 341)			
	Min–Max or	Median (Mean)	IQR	Min–Max or	Median (Mean)	IQR	*p*
Intubation time (hours)	1–1430	11 (26.3)	8	1–1400	10 (20.9)	8	**0.04**
Intensive care unit stay (days)	1–357	2 (4.2)	2	1–120	2 (3.7)	1	0.09
Hospital stay (days)	1–357	7 (11.9)	5	1–124	7 (11.5)	5	0.83

**Table 7:** Multivariate logistic regression analysis.

	Odds Ratio	CI %95	*p*
Gender male	0.915	0.660–1.270	0.59
Age	1.002	0.989–1.015	0.81
Diabetes Mellitus	0.854	0.639–1.141	0.28
COPD	0.460	0.155–0.071	0.46
Ice slush	0.002	0.500–1.368	0.94
LITA harvest	1.468	1.081–1.995	**0.01**

CI: Confidence interval, COPD: chronic obstructive pulmonary disease, LITA: left internal thoracic artery.

**Table 8:** Comparison of diaphragmatic elevations between Group C and Group D.

	Group C (Slush +) (*n* = 235) *n* (%)	Group D (Slush −) (*n* = 111) *n* (%)	
Postop 1st week diaphragmatic elevations (DE)	76 (32.3)	32 (28.8)	0.51
Postop 1st month DE	43 (18.3)	17 (15.3)	0.49
Postop 3rd month DE	26 (11.1)	11 (9.9)	0.74
Postop 6th month DE	13 (5.5)	6 (6.3)	0.54
Postop 12th month DE	4 (1.7)	2 (1.8)	0.47

## Data Availability

The data supporting this study are available upon reasonable request for research purposes.
